# Periarticular muscle status affects *in vivo* tibio-femoral joint loads after total knee arthroplasty

**DOI:** 10.3389/fbioe.2023.1075357

**Published:** 2023-03-22

**Authors:** Tobias Winkler, Louisa Bell, Alwina Bender, Adam Trepczynski, Georg N. Duda, Alexander J. D. Baur, Philipp Damm

**Affiliations:** ^1^ Berlin Institute of Health at Charité—Universitätsmedizin Berlin, Center for Musculoskeletal Biomechanics and Regeneration (Julius Wolff Institute), Berlin, Germany; ^2^ Berlin Institute of Health at Charité—Universitätsmedizin Berlin, Berlin Institute of Health Institute for Regenerative Therapies, Berlin, Germany; ^3^ Center for Musculoskeletal Surgery, Charité—Universitätsmedizin Berlin, Berlin, Germany; ^4^ Department of Radiology, Charité University Medicine Berlin, Berlin, Germany

**Keywords:** total knee arthroplasty (replacement), instrumented knee implants, periarticular muscle status, *in vivo* tibio-femoral joint loads, *in vivo* measurement

## Abstract

**Background:** Total knee arthroplasty (TKA) is a highly effective treatment for severe knee osteoarthritis that is increasingly performed in younger, more active patients. As postoperative muscular impairments may negatively affect surgical outcomes and implant longevity, functional muscle recovery gains increasing importance in meeting future patient demands. This study aimed to assess the status of periarticular muscles in the long-term follow-up after TKA and to evaluate its impact on *in vivo* tibio-femoral joint loads.

**Methods:** A case series was created, with eight patients with knee osteoarthritis. All subjects received an instrumented knee implant in unilateral TKA. Native computed tomography scans, acquired pre and postoperatively, were used to evaluate distal muscle volumes and fatty infiltration. *In vivo* tibio-femoral joint loads were measured telemetrically during standing, walking, stair climbing and chair rising and were correlated to muscle status.

**Results:** Postoperatively a reduction in fatty infiltration across all periarticular muscles was pronounced. High average peak loads acted in the tibio-femoral joint ranging from 264% during stand-to-sit activities up to 341% body weight (BW) during stair descent. Fatty infiltration of the m. quadriceps femoris and hamstrings were associated with increased tibio-femoral joint contact forces during walking (r = 0.542; 0.412 and 0.766).

**Conclusion:** The findings suggest that a fatty infiltration of periarticular muscles may lead to increased tibio-femoral joint contact forces. However, we only observed weak correlations between these parameters. Improvements in functional mobility and the restoration of a pain-free joint likely explain the observed postoperative reductions in fatty infiltration. Perioperative rehabilitation approaches targeting residual impairments in muscle quality could, contribute to reduced tibio-femoral joint loads and improved long-term outcomes of TKA. However, it has to be pointed out that the study included a small number of patients, which may limit its validity.

## Introduction

The surgical procedure of total knee arthroplasty (TKA) is increasingly performed in a younger, more active patient population, as it represents an effective treatment for end-stage knee osteoarthritis (OA). Future projections thus anticipate 55% of primary TKA procedures to be performed in patients younger than age 65 by 2030 (Kutzner et al., 2010) and an increase in the lifetime risk of revision surgery of 35% for TKA patients aged from 50 to 54 years (Bayliss et al., 2017).

The surgical outcome of TKA regarding postoperative gains in physical function and implant longevity, could be hampered by residual impairments in muscle status (Moissenet et al., 2017). Muscular weakness and fatty infiltration, foremost of the quadriceps muscle, have been described in the degenerative etiology and progression of knee OA (Kurtz et al., 2009; Alnahdi et al., 2012; Kumar et al., 2014; Kus and Yeldan, 2019). Mizner et al. () demonstrated that preoperative muscle atrophy and deficits in quadriceps strength worsen within the first month after TKA. The general focus on the quadriceps is due to the violation of the quadriceps mechanism involved in the surgical approach of TKA implantation (Kuenze et al., 2016; Moissenet et al., 2017).

A growing body of research suggests that intraoperative soft-tissue trauma may lead to muscle atrophy and fatty infiltration (Taaffe et al., 2009; von Roth et al., 2014). Atrophy and fatty degeneration are morphological characteristics of loss of functional muscle tissue, which is why they have been directly correlated to impaired muscle function and can therefore be seen as surrogate parameters of the latter (Mizner et al., 2005a; Marcus et al., 2010; Kuenze et al., 2016; Yoon et al., 2018; Kus and Yeldan, 2019; Maslaris et al., 2022). Computed tomography (CT) allows for the assessment of muscle architecture using quantitative measurements of muscle volume and voxel-based analyses of muscle density (Aubrey et al., 2014; Engelke et al., 2018). Recent evidence indicates that impairments in the joint-stabilizing function of muscles may lead to altered movement patterns and knee load distributions (Mizner and Snyder-Mackler, 2005; Farquhar et al., 2008).

Premature polyethylene wear and aseptic loosening constitute primary causes of implant failure and result from biomechanical stresses that exceed the loading capacity of the knee joint (Gallo et al., 2013). To date, the quantification of *in vivo* joint loads remains a major challenge, as instrumented knee implants are required for the direct measurement of internal joint loading conditions (D'Lima et al., 2005; Heinlein et al., 2007; Heinlein et al., 2009). Several musculoskeletal modelling tools are used to describe *in vivo* loads, but have only reached approximations (Moissenet et al., 2017; Trepczynski et al., 2018; Trinler et al., 2018). Consequently, such models can reflect the effects on the resulting joints loads *in vivo*, based on the individual muscle status, only insufficiently. However, understanding the effect of skeletal muscle architecture on *in vivo* loads acting in artificial knee joints is critical to achieving satisfactory clinical outcomes after TKA (Damm et al., 2018; Damm et al., 2019). Since musculoskeletal modelling tools could not adequately reach this aim, and real *in vivo* data on TKA loads correlated with periarticular muscle status have not yet been gathered due to the paucity of measurement implant cohorts, we established the present study to address this shortcoming. The aims of the present study were: (1) to assess long-term postoperative changes in periarticular muscle status in terms of muscle atrophy and fatty infiltration; (2) to measure the tibio-femoral joint loads *in vivo* during functional activities of daily living (ADL); and (3) to investigate the impact of the muscle status on *in vivo* tibio-femoral joint loads.

The hypothesis of this study was that an impaired periarticular muscle status results in higher *in vivo* tibio-femoral joint loads after TKA.

## Materials and methods

### Patients and study design

**FIGURE 1:**
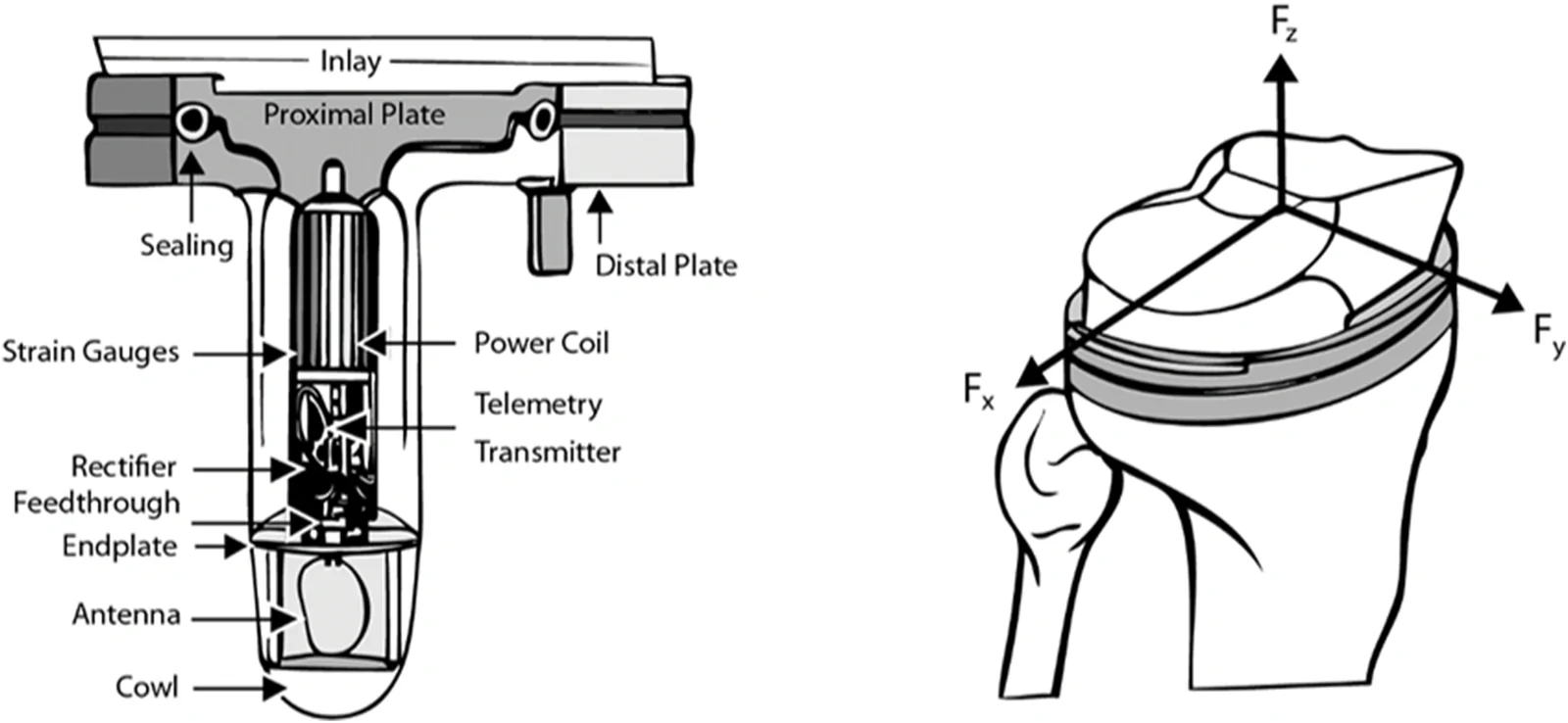
Instrumented key implant (left) and implant based coordinate system (right) based on Heinlein et al. (2007).

In the present retrospective investigation eight of nine patients [3w, 5 m; 69 ± 5 years; mean BMI 29.7 ± 4.0 kg/m^2^], who received an instrumented implant (Figure 1—left) during a primary unilateral TKA (Heinlein et al., 2007) were included. Patient characteristics are presented in Table 1. Exclusion criteria were other active implants (e.g., cardiac pacemakers), inflammatory arthritis, neurological or muscular diseases. Patients did not undergo previous contralateral TKA, except for one patient (K2L), who was asymptomatic by the time of enrolment. One patient was excluded (K1L), as CT data were not useful. The study was conducted in accordance with the Declaration of Helsinki, approved by the institutional review board (Charité—Universitätsmedizin Berlin—EA4/069/06) and registered in the German Clinical Trials Register (DRKS00000606). All subjects gave written informed consent before participation in the study and also the rights of participants were protected.

**TABLE 1:** Preoperative patient characteristics, BMI—body mass index; R—right implantation side; L—left implantation side, and pOP time of investigation.

Patient ID	K2L	K3R	K4R	K5R	K6L	K7L	K8L	K9L
Age (years)	71	70	63	60	65	74	70	75
BMI (kg/m^2^)	31.8	31.0	31.8	30.7	25.1	25.4	25.4	36.3
Sex (f/m)	m	m	f	m	f	f	m	m
pOP time (months)	48	8	38	32	32	31	27	24

### Surgery and CT protocol

TKA procedures were performed *via* the medial parapatellar approach and tibia first gap-balancing technique by two experienced surgeons at a single institution. Patients were evaluated with a comprehensive clinical examination preoperatively and postoperatively at 30 months (range: 8–48). All patients completed the same postoperative rehabilitation program and were encouraged to perform home exercises thereafter. CT scans were acquired 1 day prior and 30 months (range: 8–48) after TKA using commercially available, multi-slice scanners (Siemens Somatom Sensation 64, SOMATOM Definition Flash; both Siemens Healthcare, Erlangen, Germany and LightSpeed VCT, LightSpeed Pro 16; both GE Healthcare, Milwaukee, WI, United States). Axial CT slices were reconstructed to the narrowest standard thickness possible of 4.8 mm for all patients examined. Tube voltage was controlled with an automated dose modulation algorithm.

### Quantitative analysis of periarticular muscle status

The distal muscle volumes were evaluated by manual segmentation of muscle regions of interest (ROIs) using the Amira-Avizo Software (V6; Thermo Scientific, Waltham, MA, United States). ROIs included the four heads of the quadriceps femoris (vastus medialis, vastus lateralis, vastus intermedius, rectus femoris), the hamstrings (biceps femoris, semimembranosus, semitendinosus), gracilis and sartorius muscles. The segmented muscle volume spanned a distal thigh section of 87.7 ± 2.8 mm (18 ± 0.57 slices) and was standardized relative to the patients’ femur length, defined as distance between the top of the greater trochanter and the lower border of the lateral femoral condyle. This ensured that a comparable muscle volume was analysed in each patient, regardless of body size or leg length. Intramuscular fat content of the muscle ROIs was quantified on the four most superior contiguous axial CT-slices of the investigated thigh region with the software Image J (V1.51; U.S. National Institutes of Health, Bethesda, MD, United States). A voxel-based analysis of muscle density was performed using predefined Hounsfield unit (HU) threshold values to differentiate intramuscular fat (−190 to −30 HU) and skeletal muscle tissue (−29 to 150 HU) (Aubrey et al., 2014).

### *In vivo* measurements of tibio-femoral joint loads

Telemetric *in vivo* joint load data were obtained using a clinically proven instrumented knee implant that is based on the ultra-congruent INNEX-FIXUC system (Zimmer GmbH, Winterthur, Switzerland) (Figure 1) (Heinlein et al., 2007). Details on the design of the prosthesis and measuring units have been previously published (Graichen et al., 2007; Heinlein et al., 2007; Heinlein et al., 2009). Tibio-femoral joint loads were measured by six semi-conductor strain gages in the tibial component and transmitted to an external receiver *via* wireless transfer (Heinlein et al., 2007; Kutzner et al., 2010). The resultant joint force (F_res_) was determined from the force vectors that act *in vivo* in lateral (F_x_), superior (F_y_) and anterior directions (F_z_) from the centre of the tibial plateau, as shown in Figure 1—right (Kutzner et al., 2010). The resultant joint force values recorded during repeated movement trials were averaged and normalized to a load cycle of 100% cycle time using a time warping procedure (Bender and Bergmann, 2012). Measurements were performed postoperatively at 26 months (range: 8–45) during the activities of level walking at a self-selected speed (approx. 4 km/h), stair ascent and descent (four steps, step height 20 cm), sit-to-stand and stand-to-sit activities (chair height 45 cm) and single leg stance.

### Statistical analysis

Statistical analyses were performed using SPSS Statistics version 25 (IBM Corporation, Armonk, NY, United States). Data were expressed as means and standard deviations, unless stated otherwise. Differences between pre- and postoperative continuous and normally distributed variables in muscle status were compared with the use of paired sample *t* tests. The relationship of muscle status and selected peak values of the *in vivo* tibio-femoral joint forces (in Newton) during activities of daily living (standing, walking, stair climbing and chair rising) were assessed using Pearson’s correlations. Correlation coefficients were considered high if r ≥ 0.7 and moderate if 0.5 ≤ r < 0.7. Statistical significance was set *a priori* at *p* < 0.05.

### Data sharing

In accordance with written informed consent agreements provided by study participants, underlying *in vivo* load data files are available from the Orthoload database (http://orthoload.com/database/) using the following search criteria: Implant: Knee Joint: Level Walking; Stairs up; Stairs Down; Sit Down; Stand up. Moreover, the images of the individual muscle status can be made available upon request by the corresponding author.

## Results

### Periarticular muscle status

The analysis of postoperative distal muscle volumes showed a mean reduction of the quadriceps femoris by −9.6% (Table 2), whereby the vastus medialis accounted for 49.5% of this observed decrease. No significant alterations in distal volumes of the hamstrings, gracilis or sartorius muscles were observed, when compared to preoperative values (Table 2). The degree of fatty infiltration of the analyzed periarticular muscles (Table 2) showed a statistically significant decrease of −3.7% cross-sectional area (CSA) at the time of the 30-month postoperative follow-up. In particular, the mean decrease of intramuscular fat fraction was significant for the quadriceps (−2.8% CSA), hamstring (−4.1% CSA), gracilis (−4.9% CSA) and sartorius muscles (−3.2% CSA).

**TABLE 2:** Postoperative change of the distal muscle volume, the cross sectional area and the intramuscular fat ratio, QF–Quadriceps Femoris, RF–Rectus Femoris, VI–Vastus Intermedius, VL Vastus Lateralis, VM–Vastus Medialis, H–Hamstrings, BF–Biceps Femoris, SM–Semimembranosus, ST–Semitendinosus, PA—Pes Anserinus group, GR–Gracilis, SR–Sartorius.

Patient ID	K2L	K3R	K4R	K5R	K6L	K7L	K8L	K9L	Mean	*p*-value
**Postoperative Change of the Distal Muscle Volume [cm³]**										
**QF**	−8.2	−6.9	−11.6	−14.6	−9.2	−8.5	1.6	−15.5	−9.1	0.002
RF	1	1	−0.4	1.9	−1.1	0.6	0.3	2.0	0.6	0.130
VI	0.8	−0.2	−3.0	1.5	−2.1	−0.9	0.2	1.9	−0.2	0.696
VL	−1.8	−4.3	−8	−4	−1.2	−6.8	0	−1.3	−3.4	0.011
VM	−8.1	−3.4	−0.1	−14.2	−4.7	−1.4	1.2	−5.6	−4.5	0.035
**H**	1.2	−9.4	26.7	−10	−12.1	−12.3	6.3	−1.9	−0.2	0.972
BF	0.8	−6.9	5.1	−19.4	−8.5	−4.4	−5.4	−3.6	−5.3	0.076
SM	6.9	−2.6	17.4	8.7	−2.8	−8	11.4	1.7	4.1	0.214
ST	3.5	0.1	4.2	0.7	−0.8	0.1	0.2	0	1	0.158
**PA**	3.9	0	10	3	−2.9	−1.3	−2.6	0.8	1.4	0.391
GR	1	−0.2	4	0.9	0	−0.6	−0.2	1.3	0.8	0.179
SR	−0.5	0.1	1.9	1.5	−2.1	−0.8	−2.6	−0.6	−0.4	0.495
**All**	0.4	−1.8	2.3	−2.5	−2.6	−2.5	0.6	0.5	−0.8	0.166
**Postoperative Change of the Cross-Sectional Area [cm** ^**2**^ **]**										
**QF**	2.5	0.7	−2.9	0.1	−0.5	1.8	2.8	0.9	0.7	0.366
RF	0.9	0.9	0.5	0.6	0	0.4	0.4	0.2	0.5	0.003
VI	0.9	0.8	0.5	0.6	0.5	1.2	0.8	0.4	0.7	<0.001
VL	1.7	−1.2	−2.3	1.1	−0.1	−0.6	0.2	0.6	−0.1	0.884
VM	−1	0.2	−0.16	−2.3	−0.9	0.8	1.4	0.3	−0.5	0.318
**H**	3	−1.9	1.1	−2.8	−1.5	−0.2	0.6	−0.8	−0.3	0.650
BF	0.8	−1.8	−1	−4.5	−1	−0.9	−1.7	−1.5	1.4	0.028
SM	2.2	0.1	1.7	1.6	−0.4	0.7	2.2	0.6	1.1	0.016
ST	0.1	−0.1	0.3	0.1	−0.2	0	0.1	0.1	0	0.489
**PA**	−0.2	−0.2	0.5	0.2	−0.6	0	0.1	−0.2	0	0.727
GR	0.2	−0.1	0.3	0.2	−0.1	−0.1	0	0.1	0.1	0.239
SR	−0.4	0	−0.1	−0.1	−0.3	0.1	−0.1	−0.4	−0.2	0.050
**All**	0.6	−0.1	−0.2	−0.3	−0.3	0.2	0.4	0	0.03	0.799
**Postoperative Change of the Intramuscular Fat Ratio [%]**										
**QF**	−5.4	−5.5	−7.2	−2.	0.1	0.1	−1	−0.7	−2.8	0.041
RF	−3.1	−2.5	−3.3	−1	−1.2	−.05	−0.6	−1.3	−1.7	0.005
VI	−4.6	−0.1	−5.5	−1.3	−0.6	0	0	0.3	−1.5	0.144
VL	−5.9	−8.1	−11	−3.6	−2.6	−1.5	−1.6	2	−3.4	0.171
VM	−5.2	−4.5	−5.0	−2.7	−0.4	−1.3	−0.9	−2	−2.8	0.010
**H**	−6.7	−6.3	−9.3	−4	0.7	−1.9	−2.5	−3	−4.1	0.006
BF	−6.5	−3.7	−5.3	−2.2	2.4	−0.8	−2.6	−0.8	−2.4	0.012
SM	−6.9	−7.1	−11.6	−6.2	0.1	−2.2	−2.5	−4.5	−5.2	0.005
ST	−4.1	−16.4	−19.2	−19.5	0.8	−5.8	−1.6	−3	−8.6	0.050
**PA**	−6.5	−6.3	−9.7	−3.3	0.1	−1	−2.3	−3.6	−4.1	0.011
GR	−6.2	−3.7	−11.9	−2.3	0.1	−0.4	−6.7	−7.9	−4.9	0.020
SR	−6.8	−5.4	−6.6	−2.2	−0.1	−0.1	−1.6	−2.6	−3.2	0.018
**All**	−5.5	−5.8	−8.8	−4.6	0.4	−1.4	−2.0	−2.2	−3.7	<0.001

### *In vivo* knee joint loads

**FIGURE 2:**
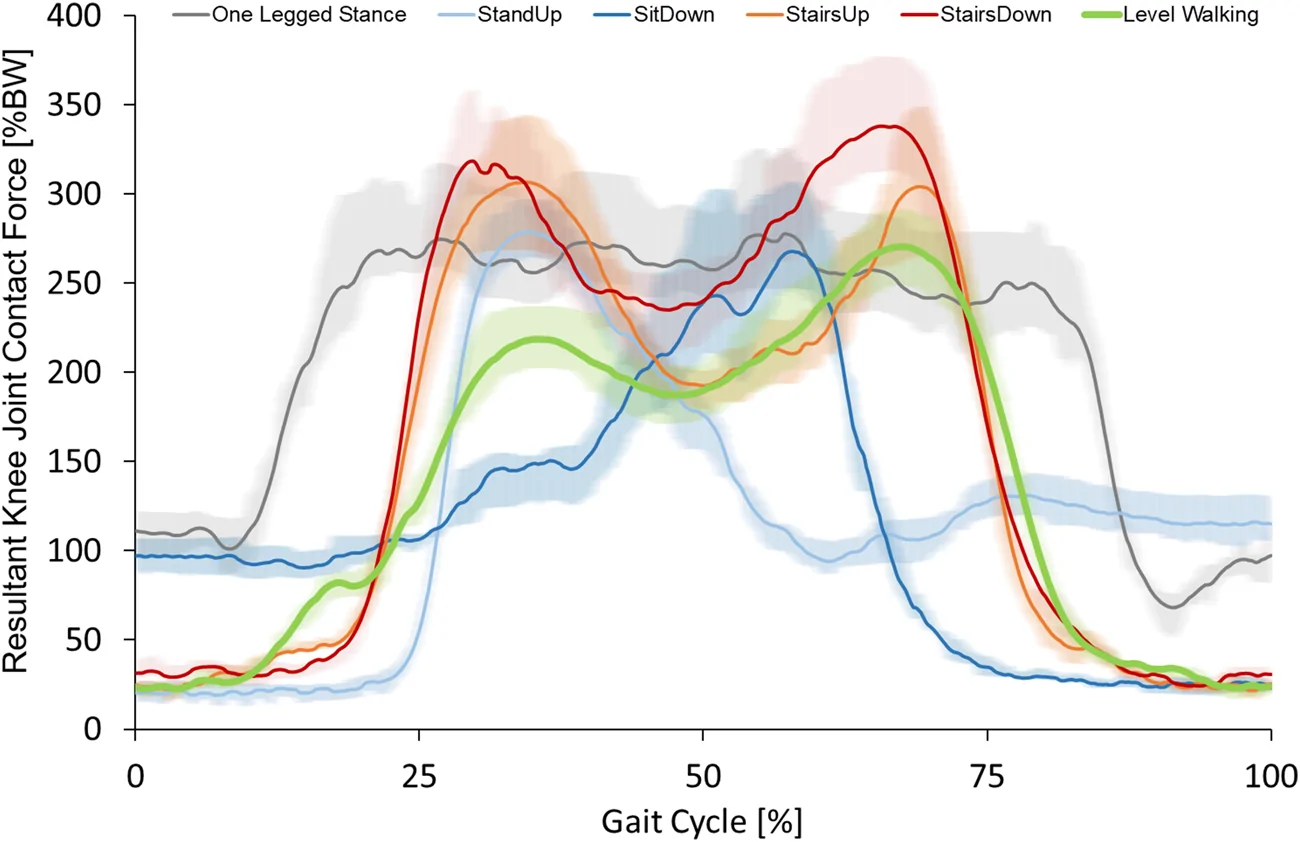
Averaged *in vivo* measured joint load patterns at the knee, during activities of daily living.

The resultant *in vivo* tibio-femoral joint loads (F_res_) were highest during stair climbing exercises with average peak values of 341% body weight (BW) during stair descent and 305% BW during stair ascent (Figure 2). In general, exercises with double-limb support showed lower peak load magnitudes with mean values of 264% and 275% BW during the activities of stand-to-sit and sit-to-stand, respectively. Substantial inter-individual differences in peak load magnitude were determined for all ADLs but were highest during stair ascent (Δ 234% BW) with values ranging from 187% BW (K9L) to 430% BW (K6L). Patient-specific load data are summarized in Table 3. Examples of the *in vivo* load and additional video data of the various activities can be accessed also *via* the public database www.orthoLoad.com (Orthoload, 2022).

**TABLE 3:** Patient-specific *in vivo* tibio-femoral joint loads in % BW [N] during functional activities. 1P—first load peak; 2P—second load peak; SD—standard deviation.

Activity	K2L	K3R	K4R	K5R	K6L	K7L	K8L	K9L	Average (SD)
Level Walking 1P	218 [1964]	202 [1936]	180 [1785]	206 [1940]	268 [2184]	249 [1691]	208 [1619]	198 [2115]	216 (29) [1904 (196)]
Level Walking 2P	253 [2282]	249 [2390]	325 [3235]	245 [2310]	291 [2372]	342 [2321]	258 [2009]	176 [1882]	267 (52) [2350 (401)]
Stairs Up 1P	344 [3108]	257 [2468]	269 [2669]	325 [3059]	348 [2835]	295 [2001]	315 [2454]	229 [2446]	298 (43) [2630 (367)]
Stairs Up 2P	349 [3154]	240 [2305]	293 [2910]	325 [3064]	430 [3501]	354 [2401]	263 [2054]	187 [2002]	305 (76) [2674 (557)]
Stairs Down 1P	417 [3766]	300 [2884]	340 [3381]	359 [3383]	366 [2977]	318 [2157]	272 [2123]	208 [2230]	323 (64) [2863 (634)]
Stairs Down 2P	392 [3536]	231 [2221]	321 [3195]	424 [3997]	374 [3045]	377 [2558]	317 [2473]	293 [3130]	341 (62) [3019 (587)]
Sit Down Max	257 [2322]	233 [2233]	248 [327]	327 [3076]	378 [3073]	264 [1791]	265 [2067]	143 [1532]	264 (68) [2320 (552)]
Stand Up Max	292 [2632]	265 [2542]	262 [2600]	297 [2800]	385 [3137]	266 [1800]	267 [2086]	164 [1750]	275 (61) [2418 (492)]
One legged Stance Max	301 [2719]	271 [2602]	285 [2836]	278 [2622]	263 [2145]	343 [2327]	362 [2823]	205 [2197]	289 (49) [2534 (275)]

### Correlation between periarticular muscle status and *in vivo* measured knee joint loads

**FIGURE 3:**
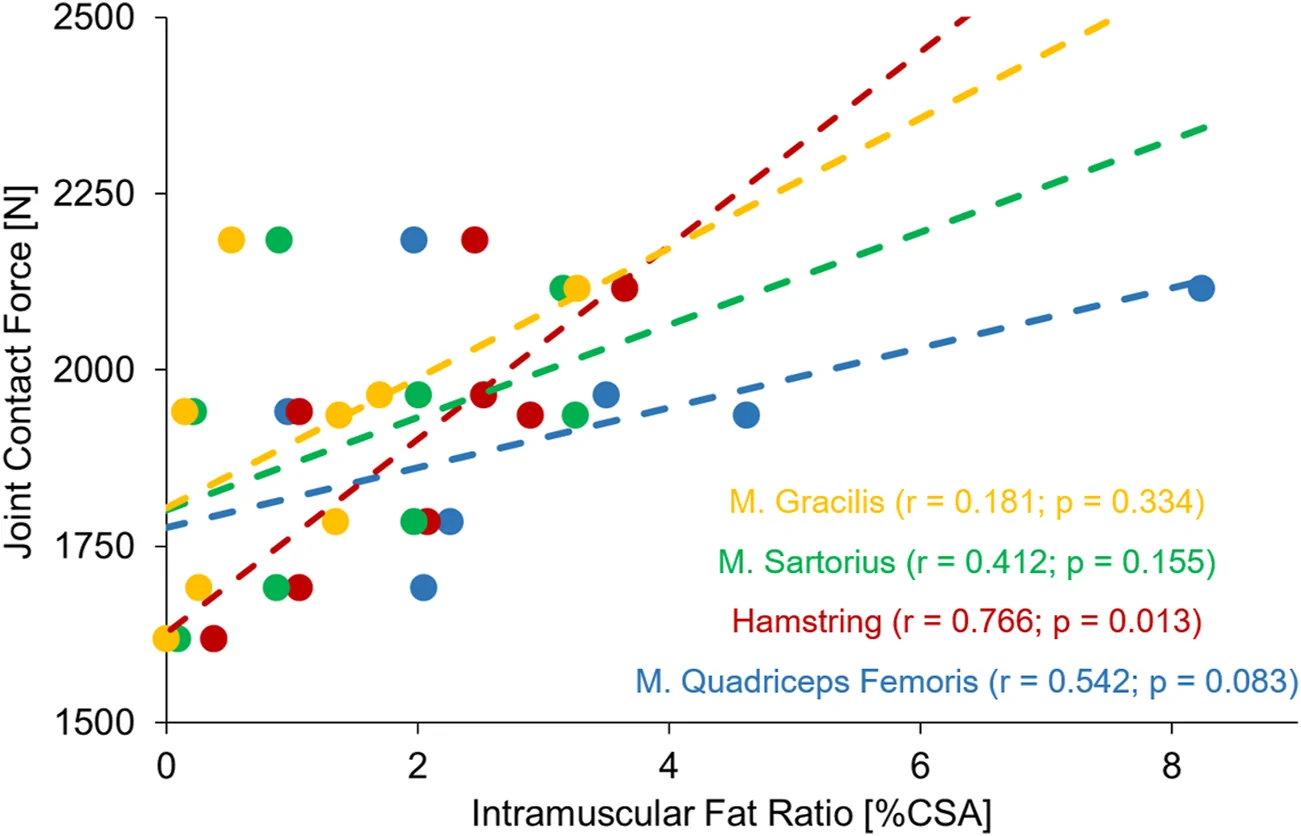
Correlation betwéen the intramusular fat ratio and the *in vivo* knee joint loading during typical activities of daily living.

To determine the suspected influence of fatty muscle degeneration on the *in vivo* acting knee contact force, the dimensions of the individual muscle volume as well as the fatty degenerations were correlated with the *in vivo* determined peak loads of each investigated activity. The results showed a significant correlation between the extent of intramuscular fat and the magnitude of peak *in vivo* tibio-femoral joint loads during level walking only (Figure 3). During the early phase of the gait cycle, during the contralateral toe-off (first peak), fatty infiltration of the vastus lateralis (r = 0.751; *p* = 0.032) and of the hamstrings (r = 0.766; *p* = 0.027), primarily of the biceps femoris (r = 0.87; *p* = 0.010), correlated significantly with increased tibio-femoral joint peak loads. Moreover, fatty infiltration of the vastus intermedius (r = 0.71, *p* = 0.050) correlated positively with increased peak forces during the late phase of the gait cycle at the contralateral heel-strike.

The correlation between distal muscle volume and peak tibio-femoral joint loads was only significant for the semitendinosus muscle (r = 0.71; *p* = 0.050) during stair descent.

## Discussion

The main aim of this study was to assess the status of the periarticular musculature and its effect on the *in vivo* tibio-femoral joint forces in the long-term follow-up after TKA. In line with the study hypothesis, our results indicate that fatty infiltration can potentially correlate with increased loadings of the tibio-femoral joint during level walking. The quadriceps muscle showed residual atrophy in the postoperative course, whereby distal muscle volume had a subordinate effect on the *in vivo* joint loading conditions.

The decrease in quadriceps distal volume by approximately 10% at 2 years after TKA corresponds with the degree of muscle atrophy in the early postoperative course as observed by Mizner et al. (2005b). Previous studies on changes in muscle size within 4 years after TKA indicate that postoperative declines in quadriceps muscle size may persist after traditional rehabilitation approaches based on resistance exercises (LaStayo et al., 2009; Valtonen et al., 2009). The decline in distal quadriceps volume could partially result from failures in volitional muscle activation (Stevens et al., 2003; Mizner et al., 2005b). A pilot study on preoperative quadriceps strengthening after TKA determined neuromuscular electrical stimulation to be a possible counter measure to limit declines in quadriceps size of 14% after 3 months of standard rehabilitation (Walls et al., 2010). Further research on the nature and timeframe of pre-habilitation concepts are warranted to establish to which extent such regimens are suitable to prevent residual muscle atrophy (Swank et al., 2011).

It is interesting to note that the vastus medialis mainly accounted for the observed distal quadriceps atrophy. This finding corroborates prior assumptions that the soft-tissue dissection during the surgical medial parapatellar approach may affect postoperative changes in muscle status (Meier et al., 2008; Kuenze et al., 2016). The present data are consistent with those of a recent study that determined a decrease in vastus medialis volume 2 years after TKA, in association with worse postoperative outcomes (Kim et al., 2021). The clinical relevance of selective vastus medialis atrophy has been highlighted by several studies suggesting an adverse effect in relation to patellofemoral pain (Pattyn et al., 2011; Wang et al., 2012) and postoperative walking speeds (Avramidis et al., 2003).

Unexpectedly, the intramuscular fat content showed an overall decrease by 4% CSA in the postoperative course that was proportionally least pronounced for the quadriceps. This outcome is likely related to improvements in functional mobility after TKA and is in line with previous investigations on the reversal of muscle fatty infiltration (Ryan et al., 2000; Taaffe et al., 2009; Trinler et al., 2018; Maslaris et al., 2022). Hence, it has been shown that the resumption of exercise after a period of detraining may improve the intramuscular lipid profile of mid-thigh muscles (Ryan et al., 2000; Taaffe et al., 2009). An analysis in sedentary, obese patients showed that intramuscular fat deposition is amenable to recovery following strict weight loss interventions and walking programs (Ryan et al., 2000). Taaffe et al. (2009) likewise determined a reduction in muscle attenuation, as a measure of fatty infiltration, after a 12-week period of resistance training by 8% and 12% for the quadriceps and hamstrings, respectively. The level of training intensity might explain the higher reductions in fatty infiltration, compared to those seen in the present patient cohort. The results underscore the importance of exercise-based rehabilitation regimens to facilitate muscle recovery (Maslaris et al., 2022).

High loads acted in the tibio-femoral joint during the functional ADLs, reaching values of up to three times body weight during stair ambulation. The measured *in vivo* joint loads are of comparable magnitude to the ones estimated by earlier *in vivo* measurements in one to six patients (Bergmann et al., 2014; D'Lima et al., 2005; Heinlein et al., 2009; Kutzner et al., 2010; Avramidis et al., 2003; Taylor et al., 2017). Peak joint loads of 290–352%BW reported in the literature during stair climbing (Bell, 2022; Bergmann et al., 2014; D'Lima et al., 2005; Heinlein et al., 2009; Kutzner et al., 2010; Taylor et al., 2017) have been explained by the complex contact mechanics and kinematics involved in this movement task (Taylor et al., 2017). The observed differences in the actual study during stair climbing (some participants experienced higher loads during stair ascent and some during stair descent) were probably caused by the individual coordination and muscle co-contraction behavior (Taaffe et al., 2009).

The only significant correlation between distal muscle volume and tibio-femoral contact forces was determined for the semitendinosus muscle during the challenging activity of stair descent. This positive association of muscle volume and mechanical joint loads could be attributed to muscle activation patterns. In studies on task-dependant muscle activation, it has been shown that amplitudes in medial hamstring activation occur during the early phase of stair descent around the time of peak knee joint loads (McFadyen and Winter 1988; Bell, 2022). Trepczynski et al. found that tibio-femoral contact forces are dominated by the forces generated by muscle contraction using musculoskeletal modelling techniques based on *in vivo* data (Trepczynski et al., 2012; Trepczynski et al., 2018). Antagonistic co-contractions of the hamstrings and the quadriceps have been related to the disease progression in knee OA (Hodges et al., 2016) and increased tibio-femoral contact forces (Trepczynski et al., 2018). No significant correlations were found for the parameter of distal muscle volume of the other periarticular muscles. Whilst this fact could result from the small sample size, it yet raises the issue of current debates on the discrepancy between muscle quantity and muscle quality (Clark and Manini, 2008; McGregor et al., 2014). Recent studies indicate that the quality and contractile properties of skeletal muscles might represent a more important determinant of physical function than anatomic parameters alone (Clark and Manini, 2008; McGregor et al., 2014).

The presented results, showing strong correlations between fatty infiltration of the m. quadriceps femoris and hamstrings and the tibio-femoral joint load, support this notion on the importance of muscle quality. This observation can be explained from a mechanical point of view as follows. If a muscle has degenerated and the joint kinematic is comparable, the patient will have to employ higher muscle activations to perform the same activities with the same joint kinematic. But the physiological limits on activation can require the additional activation of alternative muscles, which might have less advantageous lever arms relative to the joint center. Consequently the resultant joint contact force can increase.

The degree of the intramuscular fat content of both knee flexor and extensor muscles correlated with increased knee joint loads during walking. Fatty infiltration has been previously defined as a negative predictor for muscle strength and postoperative outcomes after joint replacement surgery. The ‘Health, Aging, and Body Composition’ study of 3075 elderly patients reported that thigh muscle fatty infiltration is associated with functional declines and walking difficulties (Visser et al., 2005). Damm et al. determined an association between postoperative lower lean gluteal muscle tissue and increased hip joint loads 3 months after total hip arthroplasty (Damm et al., 2018). Concerning the knee joint, it has been shown that a fatty infiltration of the vastus medialis muscle leads to higher annual tibial and patellar losses in cartilage volume (Teichtahl et al., 2015; Trinler et al., 2018). The findings indicate that the fatty replacement of contractile muscle tissue might pose a risk for implant longevity.

## Conclusion

In conclusion, although the present study indicates that a reduced fatty infiltration of periarticular thigh muscles may positively affect the loads acting in the tibio-femoral joint, we observed only weak correlations between these parameters. The observed correlations could have also been caused by secondary effects of altered muscle coordination, perhaps to compensate for weakened or lax muscles.

Postoperative decreases in fatty infiltration underscore the regenerative capacity of the periarticular musculature, whereby residual quadriceps impairments may occur. The subordinate effect of muscle atrophy on the *in vivo* joint loads also emphasizes the role of the muscles’ structural composition. Therapeutic and regenerative strategies targeted at improving periarticular muscle quality could enhance implant longevity and thus the outcome of primary TKA.

### Limitations

The study included only a small number of patients, which may limit its external validity. Moreover, the individual time point of the performed *in vivo* load measurements as well as the determination of the individual intramuscular fat ratio ranged over a long period of time. However, despite the wide range of the individual analysed postoperative time points, all participants were free of pain and with no restriction in the movements and loading abilities at the time of investigation. As a further limitation, the analysis of periarticular muscle status was only performed by segmentation of the distal portions of ipsilateral thigh muscles. An extended analysis encompassing the entire thigh muscle region could provide further insights into postoperative changes in muscle status. Non-etheless, the study presents data from the so far largest patient cohort with instrumented implants worldwide.

## Data Availability

The datasets presented in this study can be found in online repositories. The names of the repository/repositories and accession number(s) can be found below: http://orthoload.com/database/.
