# Correction: Periarticular muscle status affects *in vivo* tibio-femoral joint loads after total knee arthroplasty

**DOI:** 10.3389/fbioe.2026.1993316

**Published:** 2026-10-05

**Authors:** Tobias Winkler, Louisa Bell, Alwina Bender, Adam Trepczynski, Georg N. Duda, Alexander J. D. Baur, Philipp Damm

**Affiliations:** 1 Berlin Institute of Health at Charité—Universitätsmedizin Berlin, Center for Musculoskeletal Biomechanics and Regeneration (Julius Wolff Institute), Berlin, Germany; 2 Berlin Institute of Health at Charité—Universitätsmedizin Berlin, Berlin Institute of Health Institute for Regenerative Therapies, Berlin, Germany; 3 Center for Musculoskeletal Surgery, Charité—Universitätsmedizin Berlin, Berlin, Germany; 4 Department of Radiology, Charité University Medicine Berlin, Berlin, Germany

**Keywords:** total knee arthroplasty (replacement), instrumented knee implants, periarticular muscle status, *in vivo* tibio-femoral joint loads, *in vivo* measurement

In the **acknowledgements**, the reference is incorrect and it should be linked to Bell et al. http://dx.doi.org/10.17169/refubium-32843.

Bell L. (2022). Interrelationship of periarticular muscle status, electromyographic activity, electromechanical delay and *in vivo* knee joint loads after total knee arthroplasty, http://dx.doi.org/10.17169/refubium-32843.

The original version of this article has been updated.

